# Prognostic significance of perigastric tumor deposits in patients with primary gastric cancer

**DOI:** 10.1186/s12893-017-0280-4

**Published:** 2017-07-19

**Authors:** Shrestha Anup, Jun Lu, Chao-Hui Zheng, Ping Li, Jian-Wei Xie, Jia-Bin Wang, Jian-Xian Lin, Qi-Yue Chen, Long-Long Cao, Mi Lin, Qian Yu, Ying-Hong Yang, Chang-Ming Huang

**Affiliations:** 1 Department of Gastric Surgery, Fujian Medical University Union Hospital, Fuzhou, China; 2 Department of General Surgery, Fujian Medical University Union Hospital, Fuzhou, China; 3 Department of Pathology, Fujian Medical University Union Hospital, Fuzhou, China

**Keywords:** Gastric cancer, Perigastric tumor deposits, Survival, Prognostic significance

## Abstract

**Background:**

The presence and the prognostic significance of perigastric tumor deposits (TDs) in primary gastric cancer have not been extensively studied. The aim of this study was to evaluate the prognostic significance perigastric TDs in primary gastric cancer.

**Methods:**

From 2005 to 2010, 1250 patients underwent R0 gastrectomy at the Department of Gastric Surgery, Fujian Medical University Union Hospital, Fuzhou, China. Out of 1250 patients, 132 patients with perigastric TDs were identified. Additionally, 132 patients with staged matched gastric cancer without tumor deposits were selected as a control group.

**Results:**

Perigastric TDs were observed in 132 (10.5%) of the 1250 patients with gastric cancer who underwent R0 gastrectomy. There were 94 males (71.21%) and 38 females (28.79%) (2.47:1). The mean age was 57.21 years. Clinicopathologic characteristics between the two groups matched well. There was a significant difference in the overall survival of those with and without TDs by univariate (p<0.05) and multivariate (p < 0.05) survival analysis. The 1-, 3-and 5-year overall survival rates of patients with TDswere69.6%, 39.3%, and 24.2%, respectively, and were significantly poorer than those of the staged matched control group. There was no correlation between the number of TDs and patient survival in patients with gastric cancer (p>0.05); however, when comparing each pT tumor group with the perigastric TD group, the stage T4 survival rate was very similar to that observed in patients with TDs.

**Conclusions:**

Perigastric TDs are an independent predictive prognostic factor for gastric cancer and may be appropriately considered a form of serosal invasion. We suggest that TDs should be included in TNM staging system for better outcomes.

**Electronic supplementary material:**

The online version of this article (doi:10.1186/s12893-017-0280-4) contains supplementary material, which is available to authorized users.

## Background

Gastric cancer is an aggressive and mostly lately diagnosed disease that continues to have significant impact on cancer related death worldwide. Because most of the patients presents with advanced disease, it has a high mortality rate, making it the second leading cause of cancer-related death worldwide (http://globocan.iarc.fr/Pages/fact_sheets_cancer.aspx, [1]). It is especially prevalent in East Asia and South America and has been increasing in developing countries.

The accurate staging of gastric cancer is very important for treatment plan and prognosis. The TNM(Tumor, Lymph Nodes and Metastasis) staging system for gastric cancer is based on the infiltration depth of the primary tumor (pT), the number of metastatic lymph nodes (pN) and the presence of distant metastasis (pM) [2]. In the recent years, several other prognostic factors, such as histological type and lymphovascular invasion, intramural carcinomatosis of the lymph vessels etc., have been identified as significant and even as independent predictors for survival [3].

Tumor deposits (TDs), first recognized by Gabriel in 1935 [4],are defined as cluster of peritumoral nodules in the peritumoral adipose tissue of a primary carcinoma without histologic evidence of residual lymph node in the nodule. These may represent discontinuous spread, venous invasion with the extravascular spread, or a totally replaced lymph node [2]. The prognostic significance of tumor deposits in the colon and rectal cancers have been confirmed by several studies [2, 5–9]. Puppa et al. reported that TDs were not limited to colorectal cancers, but were also common in other tumor types including biliary duct, ovarian, gastric and pancreatic cancers [10].

The recently released 7th edition of the American Joint Committee on Cancer (AJCC) staging system for gastric cancer does not distinguish between lymph node metastasis and perigastric TDs. The AJCC staging for gastric cancer considers all perigastric metastatic nodules without evidence of residual lymph node tissue to be regional lymph node metastases [5]. The presence and the prognostic significance of TDs in gastric cancer have not been extensively studied or well documented. To date, there are only 3 series about gastric cancer tumor deposits (TDs) with prognostic implications [11–13]. The objective of this study was to investigate the prognostic significance of TDs in gastric cancer patients who underwent R0 gastrectomy and to determine whether they should be included in the TNM staging system of gastric cancer.

## Methods

This study utilized a prospectively maintained database of patients who underwent R0 gastrectomy at the Department of Gastric surgery, Fujian Medical University Union Hospital, Fuzhou, China, from January 2005 to December 2010.Of patients who underwent R0 gastrectomy for primary gastric cancer, the patients with perigastric tumor deposits were included in this study. A tumor deposit was defined as a discrete focus of tumor found in the perigastric fat or in an adjacent ligament away from the leading edge of the tumor and showing no evidence of residual lymph node tissue but within the drainage area of the primary cancer [11, 12]. An identical number of patients without TDs who underwent R0 gastrectomy during the same period and matched with same TNM stage were included as a reference group for prognostic comparison to those with TDs. No patient received neoadjuvant therapy or radiation pre-operatively. According to the Japanese Gastric Cancer Treatment Guidelines, adjuvant chemotherapy with 5-fluorouracil (5-FU)-based regimens (mostly 5-FU with cisplatin) has been administered to all patients with stage II/III GC at our institution, unless contraindicated by a patient’s condition or their refusal.

The tumor was classified according to the 7th edition AJCC staging system for gastric cancer. Clinicopathologic features such as gender, age at the time of diagnosis, differentiation type, size, location, AJCC pT category, the number of node metastases, AJCC pN category, and the type of operation were compared between the patients with and without TDs. Overall survival rates were compared between the patients with and without TDs and among patients with a different number of TDs. For each patient within the pT category, the prognosis of those with and without TDs was compared.

### Follow-up

Routine follow-up consisted of physical examination, laboratory tests, chest X-ray, abdominal and pelvic ultrasonography and computed tomography scan. Patients were followed-up every 3 months during the first year and every 6 or 12 months thereafter, for a total of 5 years. Endoscopy was performed every 1 year. All surviving patients were followed for more than 5 years. Overall survival was calculated from the date of diagnosis to last contact, date of death, or date when the survival information was collected.

### Statistical analysis

All statistical analyses and graphics were performed with the IBM SPSS version 23.0 statistical package (International Business Machines Corp., New Orchard Road Armonk, New York 10,504 914–499-1900, USA) for Windows. For comparisons of clinicopathologic characteristics between the two-propensity score-matched groups, the Chi-squared test or Fisher’s exact test were used for categorical variables as appropriate, and Student’s t-test was used for quantitative variables. Overall survival rates were determined using the Kaplan–Meier estimator, with an event being defined as death from cancer-related causes. The log-rank test was used to identify differences between the survival curves of different patient groups. In the univariate analysis, the 2-tailed Chi-square or 2-tailed t-test was used for statistical comparisons. In the multivariate analysis, Cox’s proportional hazard model was used to identify independent factors correlated with prognosis. The confidence interval (CI) method was used to compare differences in means between the predictive accuracy estimates for models that either included or did not include TDs. All *p* values were two-sided with *p* values < 0.05 considered statistically significant.

## Results

### Patients’ demographics

TDs were observed in 132 (10.5%) out of 1250 patients with gastric cancer who underwent R0 gastrectomy from January 2005 to December 2010.There were 94 male and 38 female patients with TDs (mean age of 57.21 years) and 100 male and 32 female patients without TDs (mean age of 58.64 years). Clinicopathologic characteristics of the gastric cancer patients with TDs were compared with those of the patients without TDs (Additional file 1: Table S1).

### Survival analysis

**Fig. 1:**
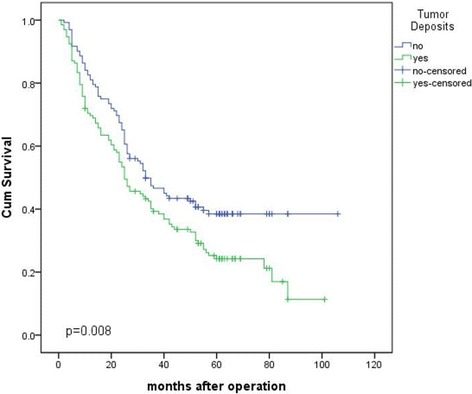
Comparison of survival curves between patients with tumor deposits (TDs) and without TDs

**Fig. 2:**
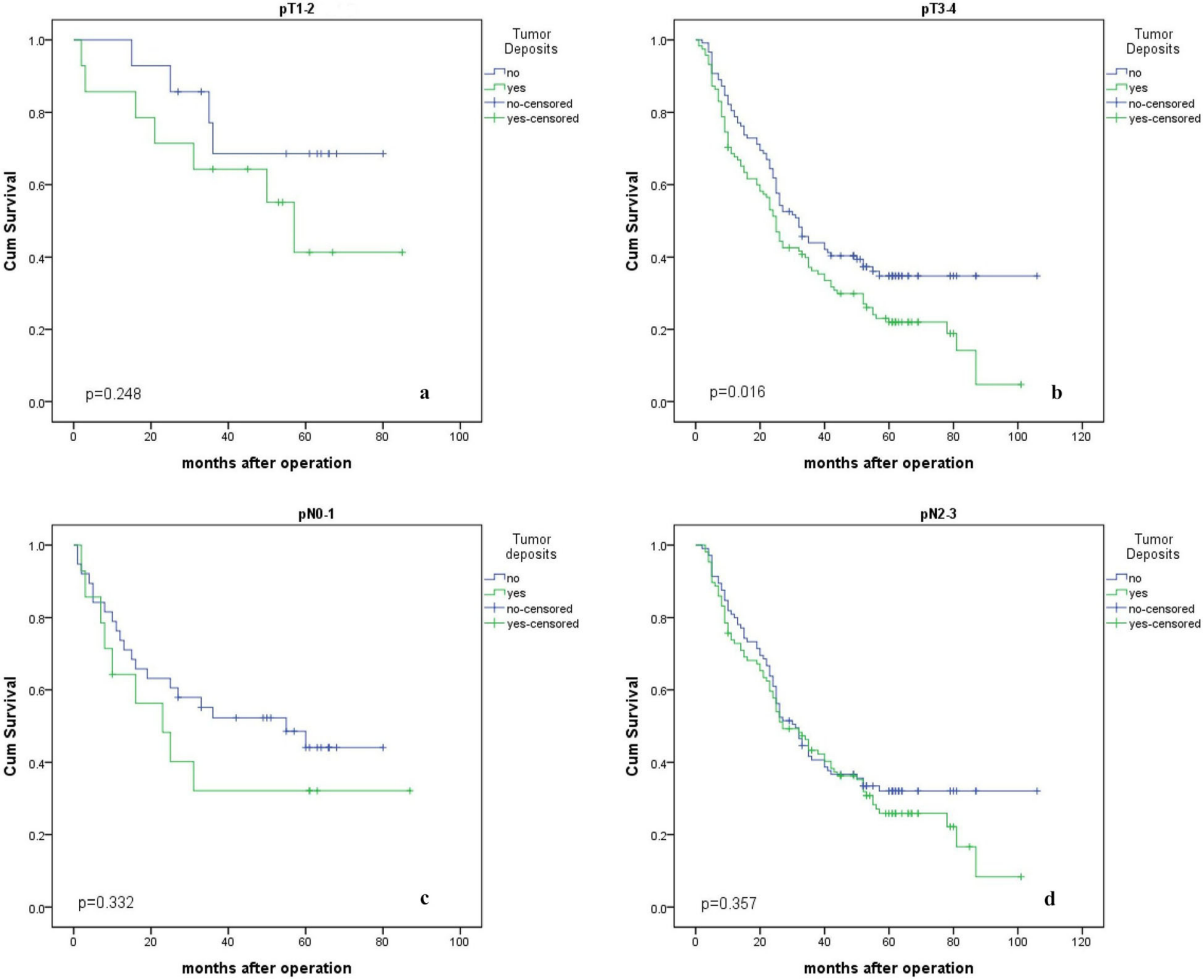
Overall survival of pT1–2 patients with TDs and without TDs (**a**); pT3–4 patients with TDs and without TDs (**b**); pN0–1 patients with TDs and without TDs (**c**); pN2-3patients with TDs and without TDs (**d**)

1. There was a significant difference between the Kaplan–Meier curves for those patients with and without TDs (*p* = 0.008) (Fig. 1). The 1-year, 3-year, and 5-year survival rates of patients with tumor deposits were 69.6%, 39.3%, and 24.2%, respectively, and those for patients without TDs were 91.7%, 46.6%, and 38.5%, respectively. We also performed a subgroup analysis regarding pT and pN stage (Fig. 2, and Additional file 2: Table S2). There was no correlation between the number of TDs and patient survival in patients with gastric cancer (*p* > 0.05) (Additional file 3: Fig. S1).
2. Univariate analysis:

Of the 8 clinicopathological variables identified by univariate analysis, the statistically significant prognostic factors for survival in all patients were tumor size (*P* = 0.014), depth of invasion (*P* ≤ 0.001), lymph node metastasis (*p* ≤ 0.001), histologic grading (*P* = 0.014), and TDs (*P* = 0.010) (Table 1).

3. Multivariate analysis:

**Table 1:** Univariate and multivariate survival analysis of the patients following operation for gastric cancer

Variable	Univariate analysis			Multivariate analysis		
	HR	95% CI	P	OR	95% CI	P
Gender(M vs F)	1.250	0.889–1.758	0.199	╱	╱	╱
Age (<60 yrs. vs ≥60 yrs)	0.942	0.702–1.265	0.692	╱	╱	╱
Location			0.351	╱	╱	╱
Middle vs Upper	0.695	0.402–1.204				
Lower vs Upper	0.958	0.616–1.489				
Whole vs Upper	1.107	0.766–1.601				
Tumor size (<5 cm vs ≥5 cm)	1.559	0.096–2.217	0.014	1.198	0.820–1.753	0.351
Histologic type (Differentiated vs Un-)	0.657	0.470–0.920	0.014	0.766	0.541–1.084	0.133
Lymph Node Metastasis			<0.001			0.009
N1 vs N0	0.497	0.292–0.847		0.770	0.432–1.372	
N2 vs N0	0.817	0.465–1.436		0.889	0.502–1.577	
N3 vs N0	0.504	0.361–0.704		0.554	0.393–0.780	
Depth of Invasion			<0.001			0.001
T2 vs T1	0.114	0.061–0.816		0.112	0.015–0.816	
T3 vs T1	0.817	0.226–0.819		0.480	0.241–0.954	
T4 vs T1	0.504	0.393–0.745		0.570	0.396–0.818	
Tumor Deposits (present vs absent)	1.475	1.098–1.981	0.010	1.411	1.029–1.936	0.033

To adjust for possible confounding baseline factors, Cox multivariate analysis was performed. Multivariate analysis of the five prognostic factors, using Cox’s proportional hazard regression model, showed that depth of invasion, lymph node metastasis, and perigastric TDs were each independent statistically significant prognostic factors (*p* < 0.05 each) (Table 1).

**Fig. 3:**
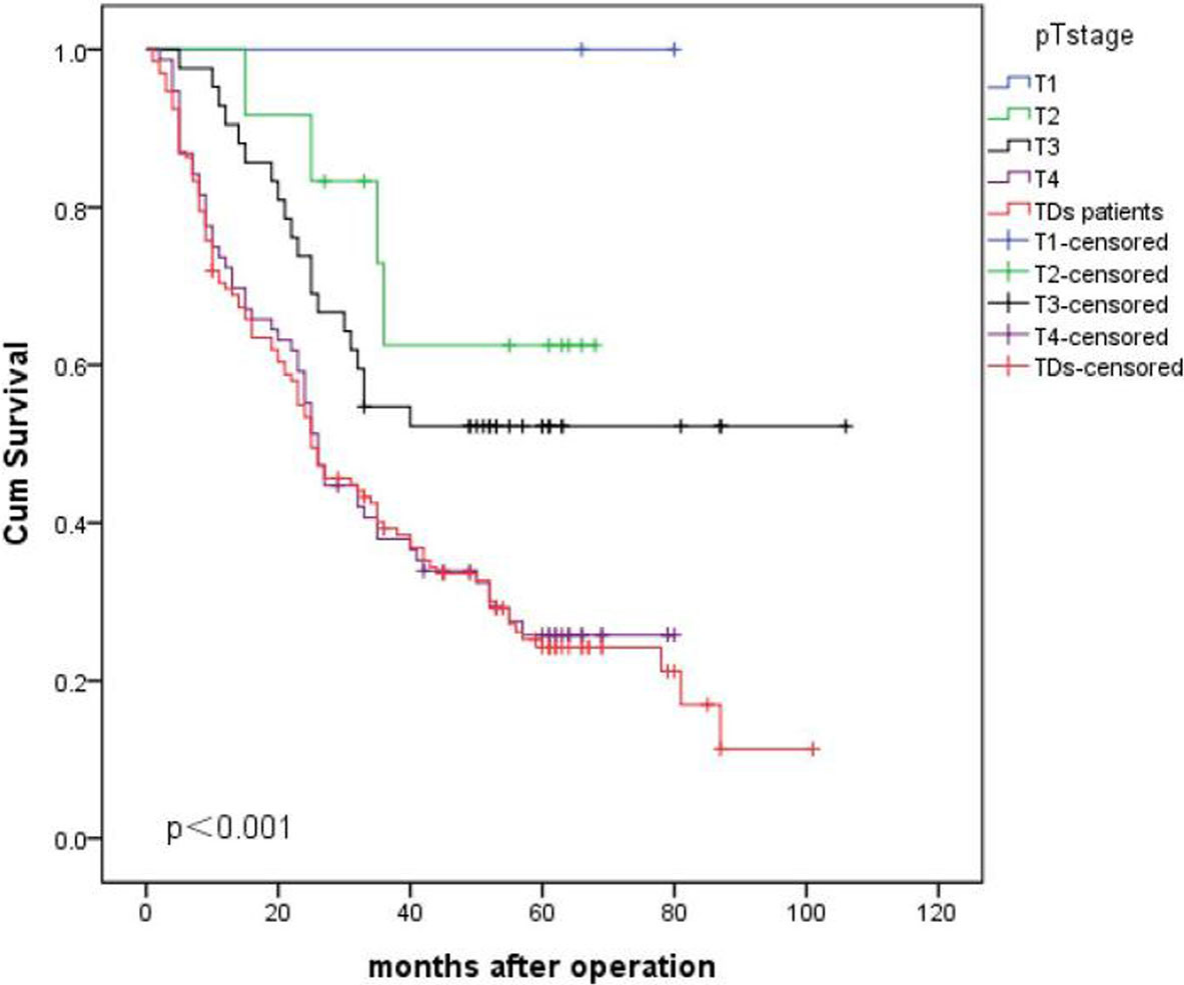
Comparison of survival curves between patients with TDs and those without TDs in pT1–4a category

4. To further elucidate the prognostic impact of TDs on gastric cancer patients who underwent R0 gastrectomy, the Kaplan-Meier survival curves were compared between patients with TDs and each pT category without TDs. The differences in the Kaplan-Meier survival curve were observed between different pT categories without TDs and with TDs. Regardless of the T stage, the patients with TDs has the worst prognosis; however, the survival rate of patients with TDs and stage T4 disease without tumor deposit was similar (Fig. 3, and Additional file 2: Table S2).

## Discussion

Even though there has been a vast improvement in the overall survival rate of patients with gastric cancer in past few decades, many questions are yet to be answered regarding the histopathological and predictive factors. Studies have demonstrated that the presence of TDs has independent prognostic value in colorectal cancer; however, only a few studies have focused on this issue in gastric cancer.

TDs are currently defined as focal clusters of tumor cells located in the serosal fat, discontinuous with the primary tumor and unassociated with a lymph node [14]. Although they are encountered in some types of adenocarcinoma, including gastric, biliary, and pancreatic [12, 13] the prognostic significance of TDs has been studied mainly in colorectal carcinomas [8, 14–20]. Currently, TDs are part of the TNM classification of colorectal carcinomas [5].

In this study, we investigate the prognostic significance of TDs in primary gastric cancer. Only 3 studies to date have been identified that focus on the prognostic value of TDs in gastric cancer. TDs were found in 17.8%, 23.9%, and 24% of cases, in the order of the date published. Sun et al. reported that TDs were more frequently observed in cancers of larger size, Borrmann type 4, in cases with lymphovascular invasion, with deeper depth of invasion and with extended lymph node metastasis, and TDs were significantly correlated to gastric cancer patients’ survival. Because of this, it might be more suitable for TDs to be treated as a form of serosal invasion. Consequently, en bloc resection of the primary carcinoma is important, and adjuvant chemotherapy should always be considered if TDs have been detected. A revised pT category, in which all cancers with TDs in pT1–4a category are incorporated into those without TDs in pT4a category, was also recommended [12].

In contrast to Sun et al., Lee et al. stated that perigastric TDs are a strong prognostic predictor in gastric cancer, and recommended TDs to be included in lymph node staging. In addition, they adopted the definition of lymph node metastasis from the AJCC (7th ed.) staging system for colorectal carcinoma and restaged gastric cancer cases after excluding TDs from being counted as lymph node metastases. In patients with the same pN stage, the presence of TDs was significantly associated with a poorer prognosis. In that study, there were two pT1 cases with TDs and four pT2 with TDs, which were only 0.9% (6 of a total of 653 patients). Therefore, TD is suggested to be less important in clinical pT staging [11].

In the most recent study, Ersen et al. reported that TDs were strongly correlated to histologic type of the primary tumor and the presence of vascular invasion. TDs were more common in intestinal type carcinomas. Compared to other studies, there was no statement about the placement of TDs as an independent prognostic factor in TMN staging [13].

In our current study, TDs were observed in 132 (10.5%) of 1250 patients with gastric cancer who underwent R0 gastrectomy. The number of TDs were as follows: 99 patients with 1 TD, 23 patients with 2 TDs, and 11 patients with more than 3 TDs. Sun et al. concluded that increasing numbers of TDs are associated with a poor prognosis [12]. In contrast to Sun et al., our results did not show any significant difference in the prognosis of patients with different numbers of TDs. There was no significant difference in the Kaplan Meier survival curve between different numbers of TDs. Belt et al. described that there was no correlation between the number of TDs and the recurrence rate in stage II patients in colorectal cancers [8], and we describe the same for the prognosis in patients with gastric cancers. It has been suggested that the lymphatic invasion is significantly associated with a poorer overall survival in node-negative gastric cancer patients [21]. But our study also suggests that the correlation between the lymphatic invasion and presence of tumor deposits was not significance. Even though our results show there are less significant role of lymphatic invasion in tumor deposits, we do believe it have significant role. Therefore, extensive detailed study should be done in the future regarding this matter.

To illustrate the effect of TDs in the prognosis of patients with gastric cancer we compared the 5-, 3-and 1-year survival rates between patients with and without TDs. We then used the Kaplan Meier curves to test the prognostic significance of perigastric TDs in gastric cancer. Our results show that the patients with perigastric TDs had a poorer prognosis than those without TDs. Multivariate Cox Regression analysis also confirmed the prognostic significance of TDs. Its hazard ratio was higher than other clinicopathologic predictive factors. This proves that TDs can be used as an independent factor for survival prognosis.

The inclusion of TDs in the TNM staging system in gastric cancer is still a matter of further study. Whether to incorporate TDs in the pN or pT category if these are to be considered in TNM staging is a matter of debate. The TDs in the colon and rectum have been well placed in the 7th edition of TNN system. The tumors with TDs are classified as the pN1c category if no lymph node metastasis has been detected. Puppa G. et al., suggested that TDs in gastric cancer are peritoneal seeding from either primary tumors or metastatic lymph nodes [10]. In this study, we placed all patients with perigastric TDs in one group regardless of their pT category, and we configured the Kaplan-Meier survival curves for the TD group and each pT category. A significant difference in overall survival was observed in between the T1, T2, T3 and TDs groups, but there was a similarity between the T4 and TDs groups (Fig. 3), suggesting that the prognosis of patients with perigastric TDs was as poor as patients with advanced T4 stage, regardless of the pT staging. Because of this, we suggest that perigastric TDs are included in pT staging, and patients with TDs should be treated separately from those without TDs when staging. Our results indicate that it might be more suitable for TDs to be considered as a form of serosal invasion; therefore, TDs should be treated as pT4a stage disease. The placement of TDs in the pT category is very effective and accurate in implications for patient prognosis and staging. These results also indicate that gastric cancer with TDs shows aggressive behavior and adjuvant chemotherapy should always be considered after en bloc resection of the tumor to achieve the better outcomes even for pT1 patients. Our results show that pT1 patients without TDs had good prognosis, but patients with TDs had worse prognosis, so even early stage gastric cancer with TDs should be considered as a highly-advanced disease and should be treated differently than those without TDs. According to our results, patients in the pT1 category with TDs had similar survival rate as patients in the pT4 category without TDs; therefore, patients with TDs should be treated as patients with advanced stage disease.

There were some limitations during the study. First, this is a retrospective study. Lauren classification was not available before 2007 in our database. Therefore, we can’t analyze the Lauren classification in the present study. Second, the numbers of pT1, pT2, and pT4b patients were low. Last but not the least, this single study from the east should also be verified in western patients. Further studies still need to be conducted.

## Conclusion

We drew the conclusion that the presence of TDs is a strong prognostic factor in gastric cancer and should be considered an independent predictive factor for prognosis. It should be considered in TNM classification as a pT4 disease.

## Additional files

Additional file 1: Table S1. Clinicopathologic characteristics of gastric cancer patients with and without TDs. (DOC 69 kb)

Additional file 2: Table S2. Comparison of T- and N- staging with different tumor deposits (TDs) factors in terms of their power to stratify patients according to overall survival (OS). (DOCX 17 kb)

Additional file 3: Figure S1. Comparison of survival curves among patients with different number of tumor deposits. (JPEG 26 kb)

## Abbreviations

AJCC: American Joint Committee for Cancer
pM: distant metastasis
pN: the number of metastatic lymph nodes
pT: depth of the primary tumor
TD: Tumor Deposit
TNM: Tumor, Lymph Nodes and Metastasis
