# Supplementary material for: Prognostic significance of perigastric tumor deposits in patients with primary gastric cancer
Source: BMC Surg. 2017 Jul 19;17:84. doi: 10.1186/s12893-017-0280-4 (PMC5518113; doi:10.1186/s12893-017-0280-4)
Supplement: Supplementary file 1 — Clinicopathologic characteristics of gastric cancer patients with and without TDs. (DOC 69 kb) [file 12893_2017_280_MOESM1_ESM.doc]

.

**Table 1.**Clinicopathologic characteristics of gastric cancer patients with and without TDs

| Variable | Tumor Deposit Absent | | Tumor Deposit Present | | P-value |
| --- | --- | --- | --- | --- | --- |
|  | n | % | n | % |  |
| Gender |  |  |  |  | 0.486 |
| Male | 100 | 75.7 | 94 | 71.2 |  |
| Female | 32 | 24.3 | 38 | 28.8 |  |
| Age |  |  |  |  | 0.537 |
| ≥ 60 | 63 | 47.7 | 57 | 43.2 |  |
| <60 | 69 | 52.3 | 75 | 56.8 |  |
| Histologic grade† |  |  |  |  | 0.596 |
| Differentiated | 44 | 33.3 | 39 | 29.5 |  |
| Undifferentiated | 88 | 66.6 | 93 | 70.5 |  |
| Size |  |  |  |  | <0.001 |
| ≥5cm | 78 | 59.1 | 69 | 52.3 |  |
| <5cm | 54 | 40.9% | 63 | 47.7% |  |
| Tumor Location |  |  |  |  | <0.001 |
| Upper | 16 | 12.1 | 15 | 11.4 |  |
| Middle | 27 | 20.5 | 25 | 18.9 |  |
| Lower | 54 | 40.9 | 60 | 45.4 |  |
| Whole* | 35 | 26.5 | 32 | 24.3 |  |
| Types of Operationᵠ |  |  |  |  | 1.0 |
| Total | 85 | 64.4 | 84 | 63.6 |  |
| Subtotal | 47 | 35.6 | 48 | 36.4 |  |
| Depth of Invasion |  |  |  |  | <0.001 |
| T1 | 2 | 1.5 | 4 | 3.0 |  |
| T2 | 12 | 9.1 | 10 | 7.6 |  |
| T3 | 42 | 31.8 | 37 | 28.0 |  |
| T4a | 73 | 55.3 | 77 | 58.4 |  |
| T4b | 3 | 2.3 | 4 | 3.0 |  |
| Lymph Node Metastasis |  |  |  |  | <0.001 |
| N0 | 16 | 12.1 | 16 | 12.1 |  |
| N1 | 11 | 8.3 | 9 | 6.8 |  |
| N2 | 46 | 34.9 | 53 | 40.2 |  |
| N3 | 59 | 44.7 | 54 | 40.9 |  |

†Graded according to Japanese classification of Gastric Carcinoma.

Differentiated , papillary or well/moderately differentiated tubular adenocarcinoma;

Undifferentiated, poorly differentiated, or mucinous adenocarcinoma or signet-ring cell carcinoma

*Whole, the location of tumor was more than 2 areas

ᵠ Total gastrectomy

Subtotal, subtotal gastrectomy(including proximal subtotal gastrectomy and distal subtotal gastrectomy )
