# Supplementary material for: Prognostic significance of perigastric tumor deposits in patients with primary gastric cancer
Source: BMC Surg. 2017 Jul 19;17:84. doi: 10.1186/s12893-017-0280-4 (PMC5518113; doi:10.1186/s12893-017-0280-4)
Supplement: Supplementary file 2 — Comparison of T- and N- staging with different tumor deposits (TDs) factors in terms of their power to stratify patients according to overall survival (OS). (DOCX 17 kb) [file 12893_2017_280_MOESM2_ESM.docx]

Supplementary Table2 Comparison of T- and N- staging with different tumor deposits (TDs) factors in terms of their power to stratify patients according to overall survival (OS)

| Variable | n | 5-year OS(%) | P |
| --- | --- | --- | --- |
| pT1-2 |  |  | 0.248 |
| With Tumor Deposits (TDs) | 14 | 41.3 |  |
| Without TDs | 14 | 68.6 |  |
| pT3-4 |  |  | 0.016 |
| With TDs | 118 | 23.0 |  |
| Without TDs | 118 | 34.8 |  |
| pN0-1 |  |  | 0.332 |
| With TDs | 14 | 32.1 |  |
| Without TDs | 38 | 44.1 |  |
| pN2-3 |  |  | 0.357 |
| With TDs | 118 | 25.9 |  |
| Without TDs | 94 | 32.1 |  |
| pT stage and TDs |  |  | ＜0.001 |
| pT1 | 2 | 100 |  |
| pT2 | 12 | 62.5 |  |
| pT3 | 42 | 52.3 |  |
| pT4 | 76 | 25.8 |  |
| Patients with TDs | 132 | 24.2 |  |
