# Supplementary figures and images for: Prognostic significance of perigastric tumor deposits in patients with primary gastric cancer
Source: BMC Surg. 2017 Jul 19;17:84. doi: 10.1186/s12893-017-0280-4 (PMC5518113; doi:10.1186/s12893-017-0280-4)

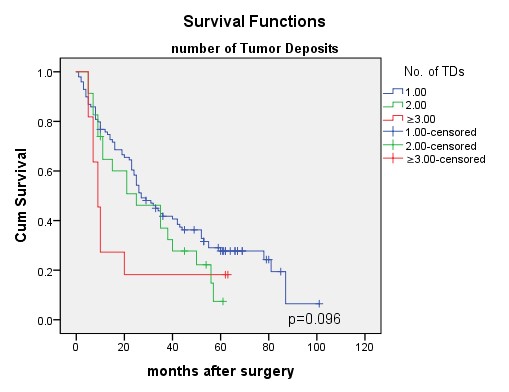

Supplement: Supplementary file 3 — Comparison of survival curves among patients with different number of tumor deposits. (JPEG 26 kb) [file 12893_2017_280_MOESM3_ESM.jpg]
